# The prognostic value of FET PET at radiotherapy planning in newly diagnosed glioblastoma

**DOI:** 10.1007/s00259-016-3494-2

**Published:** 2016-08-23

**Authors:** Sidsel Højklint Poulsen, Thomas Urup, Kirsten Grunnet, Ib Jarle Christensen, Vibeke Andrée Larsen, Michael Lundemann Jensen, Per Munck af Rosenschöld, Hans Skovgaard Poulsen, Ian Law

**Affiliations:** 1 Department of Radiation Biology, The Finsen Center, Rigshospitalet, Section 6321, Rigshospitalet, Blegdamsvej 9, DK-2100 Copenhagen, Denmark; 2 Department of Clinical Physiology, Nuclear Medicine and PET, Center of Diagnostic Investigation, Rigshospitalet, Blegdamsvej 9, DK-2100 Copenhagen, Denmark; 3 Department of Oncology, The Finsen Center, Rigshospitalet, Blegdamsvej 9, DK-2100 Copenhagen, Denmark; 4 Section of Radiotherapy, The Finsen Center, Rigshospitalet, Blegdamsvej 9, DK-2100 Copenhagen, Denmark; 5 Laboratory of Gastroenterology, University of Copenhagen, Hvidovre Hospital, Kettegård Allé 30, DK-2650 Copenhagen, Denmark; 6 Department of Radiology, Center of Diagnostic Investigation, Rigshospitalet, Blegdamsvej 9, DK-2100 Copenhagen, Denmark

**Keywords:** Glioblastoma, FET PET, Biomarker, Prognostic index, Radiation therapy

## Abstract

**Background:**

Glioblastoma patients show a great variability in progression free survival (PFS) and overall survival (OS). To gain additional pretherapeutic information, we explored the potential of *O*-(2-^18^F-fluoroethyl)-L-tyrosine (FET) PET as an independent prognostic biomarker.

**Methods:**

We retrospectively analyzed 146 consecutively treated, newly diagnosed glioblastoma patients. All patients were treated with temozolomide and radiation therapy (RT). CT/MR and FET PET scans were obtained postoperatively for RT planning. We used Cox proportional hazards models with OS and PFS as endpoints, to test the prognostic value of FET PET biological tumor volume (BTV).

**Results:**

Median follow-up time was 14 months, and median OS and PFS were 16.5 and 6.5 months, respectively. In the multivariate analysis, increasing BTV (HR = 1.17, *P* < 0.001), poor performance status (HR = 2.35, *P* < 0.001), O(6)-methylguanine-DNA methyltransferase protein status (HR = 1.61, *P* = 0.024) and higher age (HR = 1.32, *P* = 0.013) were independent prognostic factors of poor OS. For poor PFS, only increasing BTV (HR = 1.18; *P* = 0.002) was prognostic. A prognostic index for OS was created based on the identified prognostic factors.

**Conclusion:**

Large BTV on FET PET is an independent prognostic factor of poor OS and PFS in glioblastoma patients. With the introduction of FET PET, we obtain a prognostic index that can help in glioblastoma treatment planning.

**Electronic supplementary material:**

The online version of this article (doi:10.1007/s00259-016-3494-2) contains supplementary material which is available to authorized users.

## Introduction

Glioblastoma is the most common and aggressive primary brain tumor in adults. Despite standard treatment consisting of maximal surgical resection, radiation and chemotherapy with temozolomide, the prognosis is poor, with a median overall survival (OS) of less than 15 months and 5-year OS of less than 10 % [1]. OS and progression free survival (PFS) vary greatly among glioblastoma patients and less than a third of patients complete the full 6 cycles of adjuvant temozolomide treatment [2]. Given that the chemo-radiation treatment time constitutes a considerable fraction of the expected OS for most patients, it is of great interest to stratify patients by the identification of prognostic and predictive biomarkers. For instance, a recent study found that in elderly and frail patients, a short-course radiation therapy or temozolomide alone represent a viable alternative. Further, for elderly patients, the standard 6-week radiotherapy regimen seemed to be associated with substantial risk of morbidity and discontinuation [3]. Former studies on prognostic factors for glioblastoma patients treated with standard therapy suggests that age, O(6)-methylguanine-DNA methyltransferase (MGMT) protein status, use of corticosteroids and performance status are associated with OS [2, 4, 5]. Mode of surgical resection has also been suggested as a prognostic factor indicating that increasing postoperative residual tumor volume is associated with poor prognosis.[1]

Recently, the response assessment in neurooncology (RANO) working group has presented their recommendations for the clinical use of PET scans for diagnosis and treatment evaluation in the clinical management of gliomas [6]. One of the most promising radiotracers for glioblastoma evaluation is O-(2–18 F-fluoroethyl)-L-tyrosine (FET) with an excellent tumor-to-background contrast [7]. Studies have shown that the FET PET-defined tumor volume, termed biological tumor volume (BTV), reflects brain tumor tissue more accurately than morphological imaging techniques like magnetic resonance imaging (MRI) [8, 9]. Recently, two prospective studies demonstrated that the absence of postoperative FET PET tracer uptake was associated with a significantly longer survival in GBM patients, while residual tumor volume on MRI was not [4, 10]. These studies, in addition to a few other small sample-sized studies [4, 5, 10, 11] indicate the value of postoperative, preirradiation tumor volume on FET PET as a prognostic factor.

Accordingly, the aim of this study was to evaluate the prognostic value of postoperative, preirradiation FET PET in a large cohort of patients treated with standard treatment in order to improve the existing prognostic models for glioblastoma patients.

## Methods

### Patients

This study retrospectively evaluated 146 glioblastoma patients diagnosed in the period September 2011 – April 2014. All patients included were histologically verified glioblastoma patients [12]. They received radiation therapy (RT) with concomitant and adjuvant temozolomide (TMZ) as first-line treatment and received a postoperative FET PET scan as part of the RT planning 2–3 weeks postoperatively. Of the glioblastoma patients diagnosed in this period, 200 patients received RT and TMZ, and of these patients, 148 received a FET PET scan. No apparent reason for the selection was found. Two patients were excluded because of significant perioperative co-morbidities, one due to meningitis and one due to a large infarction. All patients had a minimum clinical follow-up period of 1 year. The date of last follow-up was April 13^th^ 2015. Permission for data collection was given from the Danish Data Protection Agency (2006-41-6979).

### Histologic analysis

Evaluations were made on formalin-fixed paraffin-embedded tissue. Tumor tissue was classified and graded as glioblastoma according to WHO guidelines [12].

Protein status of MGMT and isocitrate dehydrogenase 1 (IDH1) were evaluated based on immunohistochemical procedures by the pathologist at time of diagnosis. Staining of more than 10 % was considered positive for MGMT protein.

### Imaging

MRI 72 h postoperatively and for RT planning was carried out 2–3 week postoperatively and with 2 days of FET PET scanning on a 1.5-Tesla MRI scanner (Siemens, Erlangen, Germany) with acquisition of standard clinical sequences including 3D T1 (MPRAGE ) pre- and post-administration of gadolinium contrast and T2 fluid attenuation inversion recovery (FLAIR) [13, 14].

FET PET and CT scanning were performed 2–3 weeks postoperatively as part of the RT planning procedure in a single session using an integrated hybrid PET/CT system scanner (Siemens Biograph mCT, Erlangen, Germany) acquiring a single static FET PET frame acquired 20 to 40 min after i.v. injection of 200 MBq FET. At our institution, a threshold with a tumor-to-background ratio (TBR) of 1.6 is used for radiotherapy planning, based on a biopsy-controlled study showing 1.6 TBR as the optimal threshold for distinguishing between tumor and non-tumor tissue in treatment naïve glioma patients [15]. However, resection may induce reactive astroglioses [16] with increased FET accumulation supporting a more conservative TBR of 2.0 and 1.8 for pretreated glioma and long-term measurement, respectively [4, 17, 18]. Since the optimal threshold for tumor delineation is uncertain in early postoperative patients, we defined the BTV based on activity above three different TBR cut-offs: 1.6, 1.8, and 2.0. The mean (TBR_mean_) and maximum (TBR_max_) TBR ratios were calculated by dividing the mean and maximum values of the lesion by the mean activity in healthy appearing gray and white matter [13, 14] (See Appendix for details).

### Treatment

Prior to radio-chemotherapy, all patients received either a stereotactic biopsy or partial or gross total surgical tumor resection. As primary postsurgical treatment, all patients received 6 weeks of concomitant RT/TMZ therapy, with TMZ 75 mg/m^2^/day and RT at a dose of 60 Gy to the planning target volume based on both MR and FET PET imaging in 30 fractions, 5 fractions per week [13, 14, 19].

To relieve neurological symptoms, a number of patients were given corticosteroids. In this study, we considered a corticosteroid dosage of less than 15 mg a day at RT/TMZ treatment initiation as phasing out and, therefore, registered it as no use of corticosteroids. Four weeks after completion of concomitant RT/TMZ therapy, patients were given up to six courses of adjuvant TMZ therapy, one course defined as TMZ for 5 days followed by 23 days without therapy. The initial course was given at a dose of 150 mg/m2/day and the remaining courses at a dose of 200 mg/m2/day. The dose was adjusted based on relevant blood tests.

### Radiation treatment planning

Treatment planning for RT was performed three dimensionally by fusing cerebral CT with a 1-mm slice thickness with baseline MRI and FET PET on the EclipseTM treatment planning system (Varian Medical Systems, Palo Alto, CA, USA). Volumes of interest were defined in agreement with International Commission on Radiation Units & Measurements Reports 50 and 62. The gross tumor volume (GTV) was defined as the contrast-enhanced tumor on postcontrast T1 image on the baseline MRI scan. The clinical target volume (CTV) was defined as the GTV + 2 cm margin, and the FET PET BTV (>1.6 B) without additional margin, if this extended outside the CTV. In a previous publication, we showed that FET PET will increase the CTV compared to CTV(MRI) in approximately 11 % of patients with glioblastoma [13]. If present, the surgical cavity was included. The PTV was defined as the CTV + 0.5 cm margin for patient setup inconsistencies. Tolerance doses for organs at risk were as described [13]. During RT, patients were also given antibiotic prophylaxis with 400 mg sulfamethoxazole and 80 mg trimethoprim 3 times per week.

To obtain a volumetric measure of the MRI contrast enhancing residual tumor within the MRI GTV as defined by an oncoradiologist, we used a three dimensional (3D) isocontouring threshold-based method on the post-contrast T1 RT planning MRI in MIRADA software (version XD3.4, Mirada Medical Ltd, New Road, Oxford, UK). This method excluded the resection cavity and all cystic/necrotic areas. Furthermore, high intensity changes related to hemorrhage or infarcts were removed with reference to the apparent diffusion coefficient MRI sequences. All volumes were defined blinded to the FET PET scanning. The residual MRI tumor volume was set to 0 mL in all patients evaluated to have gross total resection on early postoperative MRI.

### Clinical evaluation

Patients who underwent resection were evaluated by an experienced neuroradiologist (V.A.L) with a postoperative MRI scan 48 – 72 h after surgery. During treatment, patients were evaluated with MRI scans and neurological and clinical performance after two and five courses of adjuvant TMZ. After treatment, patients were followed with the same procedures every 3 months until death.

### Study endpoints

Study endpoints were OS and PFS) OS was defined as time from start of RT/TMZ treatment until death from any cause. PFS was defined as time from start of RT/TMZ until first occurrence of disease progression [20] or death.

### Statistical considerations

The Cox proportional hazards model was used for univariate and multivariate analysis of PFS and OS. Model assessment was done using cumulative sums of martingales. The BTV on FET PET and the contrast-enhancing tumor volume on T1 weighted RT planning MRI (Gd + MRIvol) were scored as continuous covariates and hazard ratios (HRs) are presented for a 10-unit (cm^3^) difference. The survival probabilities for OS at 6, 12 and 18 months as a function of the BTV were estimated from the survivor function estimate derived from the Cox analysis. To investigate correlations between the three TBR cut-off thresholds prior to entry into prognostic models, we used Pearson’s correlations. Calculations were performed using SAS (v9.4, SAS Institute, Cary, NC, USA) and R (R Development Core Team, Vienna, Austria, http://www.R-project.org). *P* values less than 0.05 were considered significant.

### Screened factors

The following factors were screened in univariate analysis: TBR_mean_, TBR_max_, BTV (1.6), BTV (1.8), BTV (2.0), multifocal disease, gender, Gd + MRIvol, frontal location, MGMT protein status, IDH1, PS, use of corticosteroids and age.

## Results

### Patient characteristics

We included 146 patients (50 women and 96 men) in the cohort. Patient’s demographics are summarized in Table 1 (for a complete presentation of patient data see online research table S1). For second-line treatment, patients received: palliative TMZ (7.5 %), bevacizumab in combination with irinotecan (30.1 %), bevacizumab in combination with lomustine (5.5 %), monotherapy with lomustine (0.7 %), various protocolled experimental treatments (9.6 %), reirradiation (2.8 %), RT and concomitant TMZ in contralateral hemisphere (0.7 %).

**Table 1:** Patient characteristics

Population (*n =* 146)	
Gender, *n* (%)	
Female	50 (34.2)
Male	96 (65.8)
Age (years), median (range)	60 (26–79)
WHO performance status, *n* (%)	
0	81 (55.5)
1	56 (38.4)
2	9 (6.2)
Glioblastoma diagnosis, *n* (%)	
Glioblastoma	143 (97.9)
Secondary glioblastoma^a^	3 (2.1)
Multifocal disease, *n* (%)	
Yes	18 (12.3)
No	128 (87.7)
Frontal location, *n* (%)	
Yes	39 (26.7)
No	107 (73.3)
Use of corticosteroids ≥ 15 mg/d, *n* (%)	
Yes	74 (50.7)
No	72 (49.3)
Corticosteroid dose (mg), median (range)	15.0 (0–112.5)
Nr series adj. temozolomide, *n* (%)	
0	10 (6.8)
1	6 (4.1)
2	41 (28.1)
3	9 (6.2)
4	7 (4.8)
5	18 (12.3)
6	53 (36.3)
> 6	2 (1.4)
MGMT protein, *n* (%)	
Positive	48 (32.9)
Negative	98 (67.1)
Gd + MRIvol (cm^3^), median (range)	8.7 (0.0–122.4)
OS (months), median	16.46
PFS (months), median	6.54
TBR_mean_, median (range)	1.92 (0.0–3.0)
TBR_max_, median (range)	2.92 (0.0–6.94)
BTV > TBR of 1.6 (cm^3^), median (range)	21.77 (0.0–154.86)
BTV > TBR of 1.8 (cm^3^), median (range)	10.53 (0.0–127.15)
BTV > TBR of 2.0 (cm^3^), median (range)	5.36 (0.0–105.52)

^a^ Prior anaplastic astrocytoma or other histology progressing to grade IV glioma

^b^ Evaluated on 48–72 h postoperative MRI

Abbreviations: MGMT = O(6)-methylguanine-DNA methyltransferase, OS = overall survival, PFS = progression free survival, TBR = tumor-to-background ratio, BTV = biological tumor volume, Gd + MRIvol = contrast-enhancing tumor volume on T1-weighted RT planning MRI

The median BTV measured with a TBR > 1.6 was 21.8 cm^3^, TBR > 1.8 was 10.5 cm^3^ and TBR > 2.0 was 5.4 cm^3^. At the end of study follow-up, 49 patients were still alive and of these, 16 patients were still alive without progression. Median follow-up time was 14 months with a median OS and PFS of 16.5 months and 6.5 months, respectively.

### Univariate analyses

In univariate analyses, factors significantly associated with poor OS (online research table S2) were larger BTV (for all three TBR’s *P* < 0.001), higher TBR_mean_ (*P* < 0.001), higher TBR_max_ (*P* < 0.001), older age (*P* = 0.002), WHO PS 1 vs. 0 (*P* < 0.001), WHO PS 2 vs. 0 (*P* < 0.001), use of corticosteroids ≥ 15 mg (*P =* 0.001), MGMT protein-positive (*P* < 0.001) and contrast-enhancing tumor volume on MRI (*P* < 0.001).

Factors associated with poor PFS were (online research table S2) larger BTV (*P* < 0.001), higher TBR_mean_ (*P* < 0.001), higher TBR_max_ (*P* < 0.001), WHO PS 1 vs. 0 (*P* = 0.004), use of corticosteroids > 15 mg (*P =* 0.04), MGMT protein-positive (*P* = 0.006). Contrast-enhancing tumor volume on MRI was not a significant prognostic factor (*P* = 0.082).

A Pearson correlations test showed a very strong correlation (*p* < 0.0001) between BTV delineated with the three different tumor thresholds. The correlations coefficients were: 1.6 vs. 1.8: r = 0.99; 1.6 vs. 2.0: r = 0.96; 1.8 vs. 2.0: r = 0.99.

A Mann–Whitney U test showed a strong correlation between the use of corticosteroids and both PS (*P* < 0.0001) and BTV (*P* = 0.005).

### Multivariate analysis

All three BTV delineated with different activity thresholds were strongly correlated and all were significantly associated with OS and PFS in univariate analyses. We found no obvious advantage of a more conservative threshold for prognostic evaluation. Thus, for the multivariate model, we used a BTV based on a TBR > 1.6 B which is already used in our standard clinical practice for RT planning. Due to a small sample size of the PS 2 group, PS 2 vs. PS 0 was insignificant when included in the multivariate model. In order to adjust for PS and to simplify the model, the two groups, PS 1 and PS 2, were pooled for the final multivariate analysis.

In the multivariate analysis (Table 2), factors associated with poor OS were larger BTV (per 10 cm^3^ HR = 1.17, 95 % CI: 1.07–1.27, *P* < 0.001), poor PS (HR = 2.35, 95 % CI: 1.54–3.59, *P* < 0.001), MGMT protein-positive (HR = 1.61, 95 % CI: 1.07–2.42, *P* = 0.024), and higher age (per decade HR = 1.32, 95 % CI: 1.06–1.63, *P* = 0.013). Gd + MRIvol was not prognostic of OS (*P* = 0.72). For PFS, only larger BTV (HR = 1.18, 95 % CI: 1.07–1.31, *P* = 0.002) was of significant prognostic value.

**Table 2:** Multivariate analyses

	Without FET PET BTV		With FET PET BTV	
	PFS HR (95 % CI)	**OS HR** (**95** % **CI**)	**PFS HR** (**95** % **CI**)	**OS HR** (**95** % **CI**)
**BTV** ^#^ (/**10 cm** ^**3**^) **on FET PET**			**1.18** (**1.07**–**1.31**) ***p*** = **0.002**	**1.17** (**1.07**–**1.27**) **p** < **0.001**
**Performance status** (**1**–**2 vs. 0**)	1.37 (0.93–2.02) *P* = 0.109	**2.1** (**1.38**–**3.18**) **P** < **0.001**	1.32 (0.90–1.94) *P* = 0.154	**2.35** (**1.54**–**3.59**) **P** < **0.001**
**Age** (**decades**)	1.07 (0.91–1.25) *P* = 0.444	**1.29** (**1.05**–**1.59**) ***P*** = **0.015**	1.09 (0.92–1.29) *P* = 0.311	**1.32** (**1.06**–**1.63**) ***P*** = **0.01**
**MGMT protein** (**positive vs. negative**)	1.38 (0.93–2.04) *P* = 0.107	**1.69** (**1.12**–**2.56**) ***P*** = **0.013**	1.31 (0.89–1.93) *P* = 0.179	**1.61** (**1.07**–**2.42**) ***P*** = **0.024**
**Gd** + **MRIvol** (/**10 cm** ^**3**^)	1.06 (0.97–1.15) *P* = 0.239	**1.15** (**1.05**–**1.25**) ***P*** = **0.003**	0.87 (0.72–1.06) *P* = 0.162	1.03 (0.89–1.19) *P* = 0.72

^#^ BTV measured above tumor-to-background ratio of 1.6

Significant parameters in bold

Abbreviations: BTV = biological tumor volume, FET PET = *O*-(2–^18^F-fluoroethyl)-L-tyrosine positron emission tomography, MGMT = O(6)-methylguanine-DNA methyltransferase, Gd + MRIvol = contrast-enhancing tumor volume on T1-weighted RT planning MRI

**Fig. 1:**
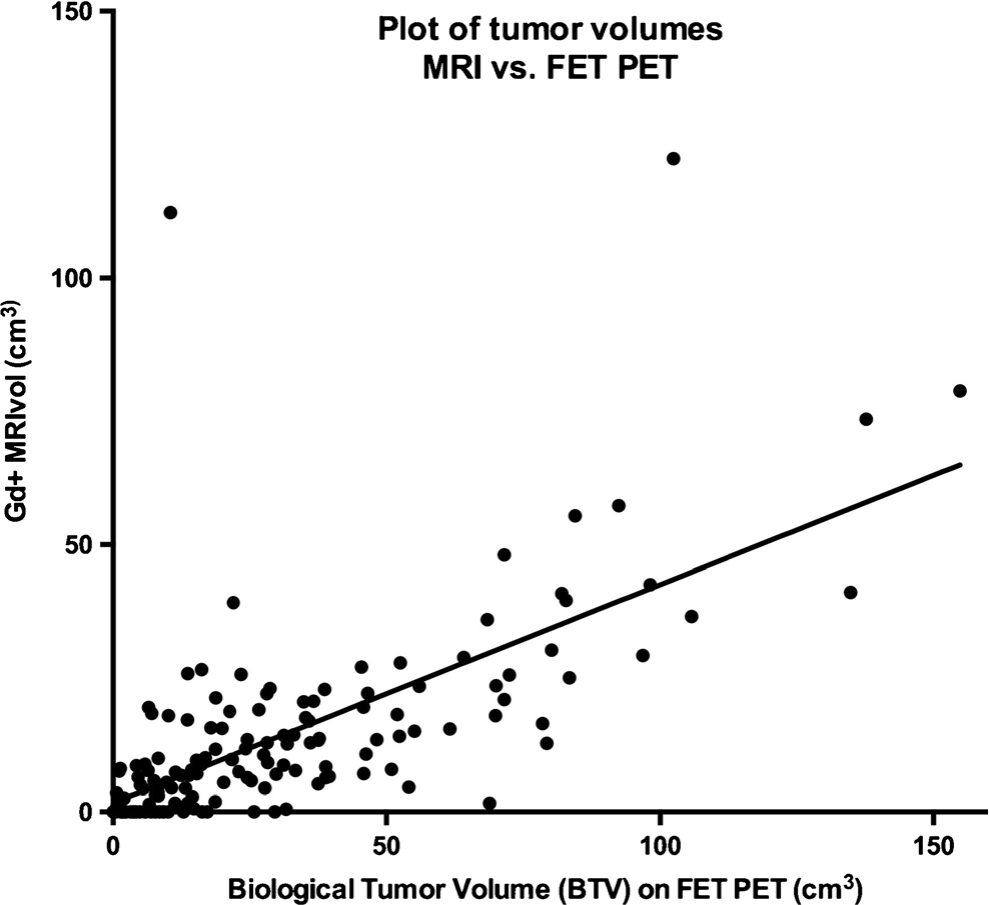
Plot of tumor volumes: contrast-enhancing tumor volume on T1 MRI and biological tumor volume (BTV) on FET PET. There is a significant correlation between contrast-enhancing tumor volume on T1 MRI (Gd + MRIvol ) and BTV on FET PET, *r* = 0.67, *r* ^*2*^ = 0.45 (*P* < 0.0001). The slope of the curve is 0.41, indicating a systematic underestimation of Gd + MRIvol compared to BTV. Volumes are in cm^3^

However, in an independent multivariate analysis without BTV, Gd + MRIvol *was* prognostic of OS (HR = 1.15, 95 % CI: 1.05–1.25*, P* = 0.003) but not of PFS (Table 2). The correlation between Gd + MRIvol and BTV was r = 0.67 (*p* < 0.0001), r^2^ = 0.45 with a slope on linear regression of 0.41 (95 % CI: 0.33–0.48; Fig. 1).

### Prognostic index

**Fig. 2:**
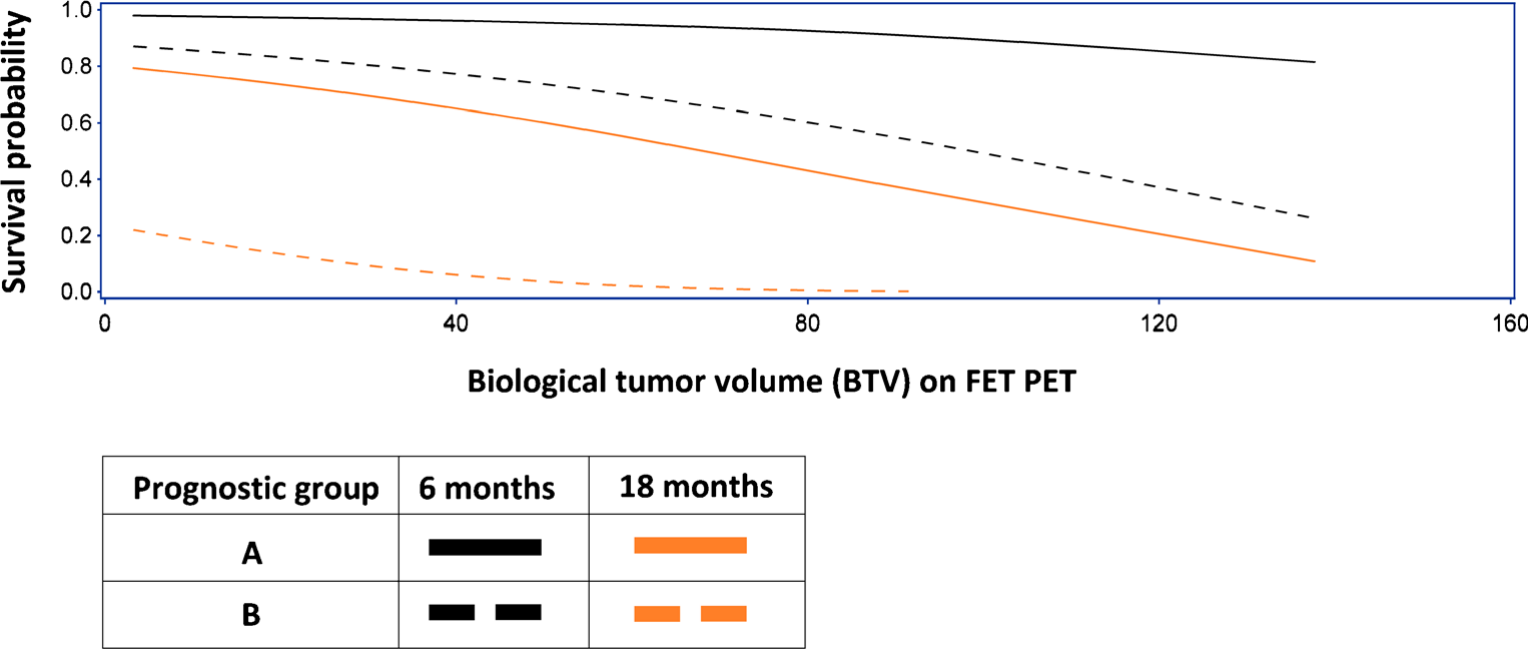
Estimated survival probability at 6 and 18 months as a function of biological tumor volume (BTV). The short-term (6 months) impact of increasing BTV glioblastoma patients is most pronounced in the poor prognostic group (**b**) and in the long term (18 months) in the good prognostic group (**a**). BTV is in cm^3^, and derived from FET PET (>1.6 B). Prognostic groups: A: age 50, MGMT protein-negative, PS 0. B: age 70, MGMT protein-positive, PS 1–2

Based on significant factors from the multivariate analysis, we defined a prognostic index model where the survival probabilities at 6, 12 and 18 months can be estimated as a function of the BTV (TBR > 1.6 B). We divided patients into prognostic groups based on age, MGMT protein status and PS. The prognostic value of BTV FET PET at 6 and 18 months in the worst and best prognostic group is visualized in Fig. 1. For all groups and 12-month survival, see online research figure S2. As seen in Fig. 2, a patient in a good prognostic group (Age 50, MGMT protein negative, PS 0) with a BTV of 10 cm^3^ has a 77 % chance at being alive at 6 months, whereas a patient in the poor prognostic group (Age 70, MGMT protein positive, PS 1-2) with at BTV of 120 cm^3^ has an 18 % chance at being alive at 6 months.

## Discussion

**Fig. 3:**
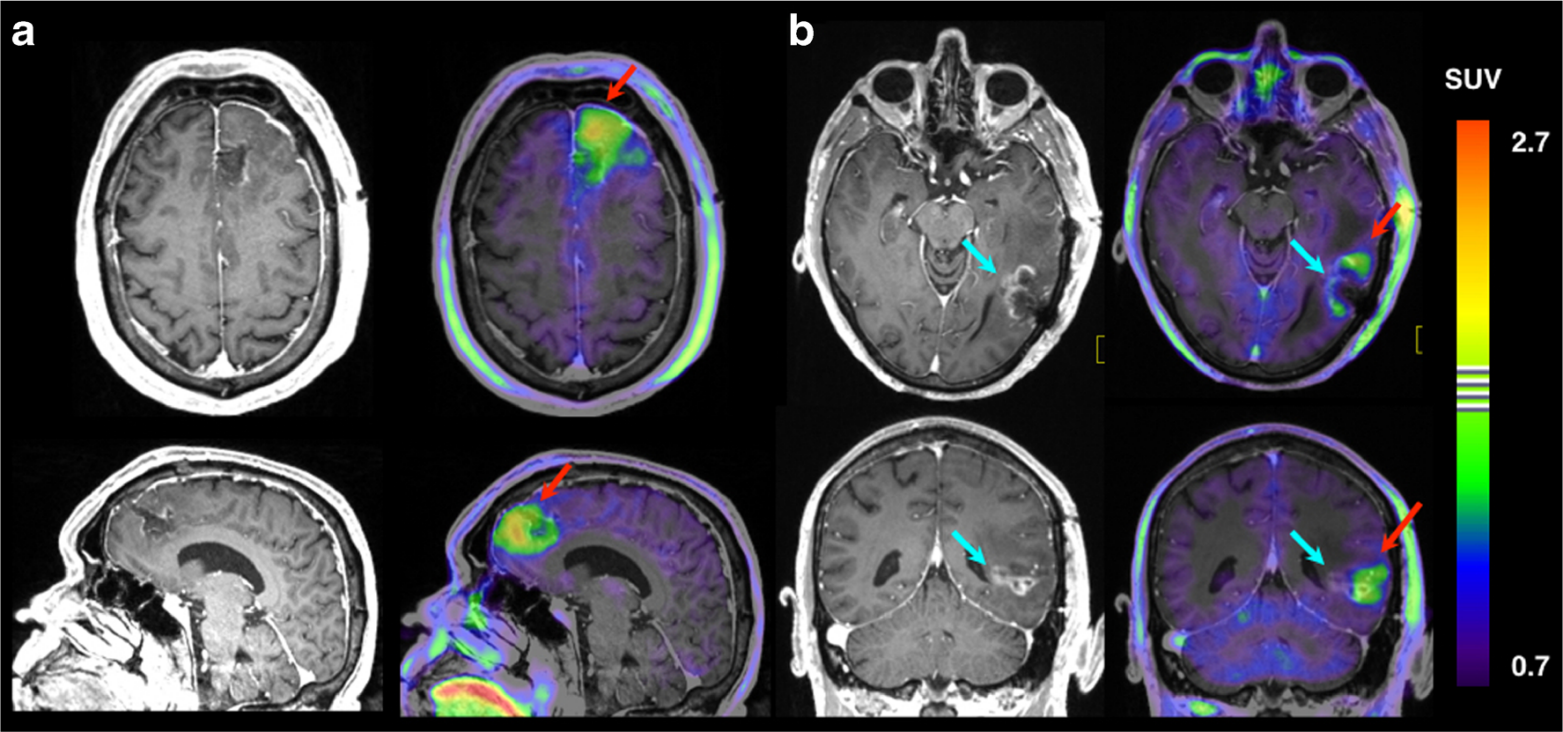
FET PET and early postoperative T1-weighted MRI. Early (48–72 h) postoperative post-contrast T1-weighted MRI alone and fused to FET PET scanning performed 2 weeks later for radiation treatment planning showing examples of conflicting tumor definitions of the two techniques. **a**. Residual metabolically active non-enhancing tumor remnant anterior to the resection cavity identified in the left frontal lobe (*red arrow*), presumably infiltrating glioblastoma. On MRI, the tumor was evaluated as gross *total* resected. Patient ID 30. Gd + MRIvol: 0 cm^3^; BTV: 26 cm^3^; OS: 13 months. See Table S1. *Top*: Transaxial section above corpus callosum. *Bottom*: Sagittal section left of midline. This patient was evaluated as *partially* resected based on a smaller contrast enhancing region (*blue arrow*) in the depth of the resection cavity in the left temporal region. Lack of metabolic activity on FET PET, however, suggests reactive changes. Areas of increased uptake is found in subcortical white matter anterior and posterior to the cavity (*red arrow*), presumably infiltrating glioblastoma. Patient ID 98. Gd + MRIvol: 3.2 cm^3^; BTV: 7.8 cm^3^; alive at 31 months follow-up. See Table S1. *Top*: Transaxial section at the level of mesencephalon. *Bottom*: Mid-cerebellar coronal section

This retrospective study is, to date, the largest standardized published series evaluating the prognostic value of FET PET scanning postoperatively in a multivariate setting in glioblastoma patients. We found that BTV, regardless of which of three different activity cut-off thresholds was used, together with PS, MGMT protein and age were strong independent prognostic factors for OS. For poor PFS, increasing BTV and poor performance status were prognostic. The rationale behind the higher thresholds applied in previous studies [4, 11] adhere to the increased non-specific FET uptake in regional reactive astrogliosis following surgical trauma [21–23], but could also underestimate clinically significant gliomatose infiltration (Fig. 3). The strong correlation between the three BTV thresholds indicates that for a statistical and prognostical evaluation, there is no clear advantage of one threshold over another, probably reflecting the above trade-off between sensitivity and specificity for tumor tissue. A previous smaller FET PET study similarly did not find any prognostic superiority of different thresholds [11]. For practical reasons, we chose TBR > 1.6 B for the multivariate analysis since it is already implemented in the clinic for standard RT planning at our institution, where tumor sensitivity is a priority. Previous studies indicate the use of different thresholds at different stages of tumor evaluation whether in primary diagnosis, RT planning, or in assessment of recurrence or response [15, 24, 25].

TBR_mean_ and TBR_max_ were associated with OS and PFS in the univariate analysis, but failed to predict survival in the multivariate model, similar to a previous study [4]. This is not surprising given that FET is not metabolically fixated in tissue and the kinetics differ between tumors [4]. As we acquire images only late in the time-activity curve, these different kinetics will increase the variability in TBR_mean_ and TBR_max_ and influence the prognostic value.

Former studies on prognostic factors for glioblastoma patients treated with standard therapy suggests that age, MGMT protein status, use of corticosteroids and performance status are associated with OS [2, 4, 5]. A FET PET study identified postoperative BTV as the most important independent imaging biomarker prognostic for both PSF and OS irrespective of surgical mode [4]. Our study confirms BTV, age, MGMT protein status and performance status. In our model, positivity for MGMT protein was found prognostic of poor OS. In this retrospective study, polymerase chain reaction (PCR) of MGMT promotor methylation status on the DNA level was not performed. The evaluation of MGMT protein in the tumor cells using immunohistochemistry (IHC) was the diagnostic standard in glioblastoma pathology during the observation period. Previously, we have demonstrated a good agreement between MGMT promotor methylation and MGMT protein status in a study of 151 glioblastoma patients from our institution and, therefore, we find MGMT protein a reliable surrogate marker for MGMT promotor methylation, at least in our hands, and that our data and interpretation would not change should we reanalyze the material using the PCR technique [26].

Based on the significant factors in the multivariate model, we formulated a prognostic index using Cox modeling for OS. The prognostic index can be used in clinical practice to estimate survival probability of the individual patient at 6, 12 and 18 months as a function of BTV (Fig. 1, online research figure S2). The model underlines the relative sensitivities of increasing BTV in different patient populations revealing a profound dissociation. Increasing BTV has a marked short-term negative impact on estimated survival probability in the poor prognostic group. However, for the good prognostic group, the impact of BTV is only slight on the short term, and becomes clinically significant on the 18-month estimated survival probability. The described prognostic model can potentially be a valuable tool in the clinical decision process, tailoring the optimal therapeutic strategy for the individual glioblastoma patient. Thus, it could be used to identify unfortunate patients with a life expectancy of less than 6 months, where standard treatment can be considered futile. Conversely, it could identify potential long-time survivors, where preservation of cognitive function and quality of life is given high priority and is reflected in the choice of therapy. It follows that the model could be used to reduce the risk of unnecessary side effects in both patient profiles. It should be noted that the model in its present form should be considered hypothetical and validation in an independent cohort prior to practical use is a prerequisite.

We found that larger BTV was prognostic of poor PFS as in previous studies [4, 11]. PFS is only marginally confounded by second-line therapy, and has the benefit of offering an earlier assessment and higher statistical power at the time of analysis. A prolonged PFS by itself may have a clinically significant beneficial impact on overall function and quality of life for the glioblastoma patient given the infiltrative and destructive nature of the disease. A recent large metaanalysis of 91 unique glioblastoma trials has shown that PFS and OS are strongly correlated, indicating that PFS may be an appropriate surrogate endpoint for OS [27].

One limitation was the use of the Macdonald criteria, but it has been shown that PFS evaluated by both RANO and Macdonald were associated with OS [28].

The prognostic value of postoperative BTV found in this and in other FET PET studies [4, 10, 11] repeatedly underlines the importance of maximal tumor resection [10, 29, 30]. We found a strong correlation between Gd + MRIvol and the BTV (Fig. 2) with a slope significantly lower than unity, indicating a general underestimation of tumor volumes based on Gd + MRIvol compared to BTV. In multivariate analysis, Gd + MRIvol did have independent prognostic value, providing BTV was not included, in which case it was lost. Only BTV was prognostic for PFS, while Gd + MRIvol was not in any combination emphasizing BTV as the more important prognostic factor. Extending the MRI tumor volume by including non-contrast-enhancing changes on T2-weighted sequences will not be practical on MRI 2–3 week postoperatively because of reactive changes, including oedema, ischemia, and gliosis, limiting the value in evaluating the extent of surgical resection and local tumor control.

## Conclusion

BTV is a strong independent prognostic factor for OS and PFS for glioblastoma patients treated with surgery and radio-chemotherapy. A model including BTV, MGMT protein status, PS and age can estimate the probabilities of OS for glioblastoma patients and can, if validated, be implemented in the clinical practice for treatment stratification.

### Electronic supplementary material

Below is the link to the electronic supplementary material.

ESM 1 (DOCX 714 kb)

## Appendix

### PET scanning

FET PET and CT scanning was performed as part of the RT planning procedure in a single session using an integrated hybrid PET/CT system scanner (Siemens Biograph mCT, Erlangen, Germany) with the patient’s head positioned in a moulded plastic net fixation device directly attached to a scanner flat-top cradle. A single static FET PET frame was acquired 20 to 40 min after i.v. injection of 200 MBq FET. For all images, default random, scatter, and dead time correction and low-dose CT-based attenuation correction was applied. Image reconstruction was performed using OSEM 3D (4 iterations, 16 subsets with a matrix size of 336 x 336 x 74 [0.8 x 0.8 x 3 mm voxel size]). Images were filtered with a 5-mm FWHM Gaussian filter.

The FET PET image was co-registered to the T1-weighted post-gadolinium contrast RT planning MRI. A 3D crescent-shaped background (B) region of interest (ROI), encompassing the activity above 70 % of maximum, was delineated in healthy appearing gray and white matter of 4 contiguous brain slices above the insula in the contralateral hemisphere to the tumor. When delineating a tumor on a FET PET scan, the exact threshold for the metabolic activity that distinguishes between tumor tissue and unspecific postoperative reactive changes is not well defined. At our institution, a threshold with a tumor-to-background ratio (TBR) of 1.6 is used for radiotherapy planning, based on a preoperative biopsy-controlled study showing 1.6 TBR as the optimal threshold for distinguishing between tumor and non-tumor tissue [31]. Another study found a TBR of 2.0 as the best threshold for pretreated glioma [32, 33]. A third study used a TBR of 1.8 for long-term measurement [34]. Therefore, the BTV derived from FET PET was auto-contoured in 3D, defining tumor tissue at a threshold of above 1.6, 1.8 and 2.0 of the mean standardized uptake value (SUV) in the background ROI (Syngo-TrueD, Siemens). TBRmean and TBRmax were calculated by dividing the mean and maximum values of the lesion by the mean SUV B.

## Abbreviations

BTV: Biological tumor volume
CTV: Clinical target volume
FET: *O*-(2-^18^F-fluoroethyl)-L-tyrosine
FLAIR: Fluid attenuation inversion recovery
Gd + MRIvol: Contrast enhancing tumor volume on T1-weighted RT planning MRI
GTV: Gross tumor volume
IDH1: Isocitrate dehydrogenase 1
MGMT: O(6)-methylguanine-DNA methyltransferase
PET: Positron emission tomography
PS: Performance status
PTV: Planning target volume
RANO: Response assessment in neurooncology
RT: Radiation therapy
TBR: Tumor-to-background ratio
TBRmean: Mean tumor-to-background ratio
TBRmax: Maximum tumor-to-background ratio
TMZ: Temozolomide
