# Supplementary material for: The prognostic value of FET PET at radiotherapy planning in newly diagnosed glioblastoma
Source: Eur J Nucl Med Mol Imaging. 2016 Aug 23;44(3):373–81. doi: 10.1007/s00259-016-3494-2 (PMC5281673; doi:10.1007/s00259-016-3494-2)
Supplement: Supplementary file 1 — (DOCX 714 kb) [file 259_2016_3494_MOESM1_ESM.docx]

**The Prognostic Value of FET PET at Radiotherapy Planning in Newly Diagnosed Glioblastoma**

**Authors:**

Sidsel Højklint Poulsen, Thomas Urup, Kirsten Grunnet, Ib Jarle Christensen, Vibeke Andrée Larsen, Michael Lundemann Jensen, Per Munck af Rosenschöld, Hans Skovgaard Poulsen, and Ian Law

**Affiliations:**

Department of Radiation Biology, The Finsen Center, Rigshospitalet, Blegdamsvej 9, DK-2100 Copenhagen, Denmark (S.H.P., T.U., K.G., H.S.P.);

Department of Oncology, The Finsen Center, Rigshospitalet, Blegdamsvej 9, DK-2100 Copenhagen, Denmark (S.H.P., T.U., K.G., M.L.J., P.M.R., H.S.P.);

Section of Radiotherapy, The Finsen Center, Rigshospitalet, Blegdamsvej 9, DK-2100 Copenhagen, Denmark (M.L.J., P.M.R.)

Laboratory of Gastroenterology, University of Copenhagen, Hvidovre Hospital, ​Kettegård Allé 30, DK-2650 Copenhagen, Denmark (I.J.C.);

Department of Radiology, Center of Diagnostic Investigation, Rigshospitalet, Blegdamsvej 9, DK-2100 Copenhagen, Denmark (V.A.L.);

Department of Clinical Physiology, Nuclear Medicine and PET, Center of Diagnostic Investigation, Rigshospitalet, Blegdamsvej 9, DK-2100 Copenhagen, Denmark (S.H.P., I.L)

**Running title:**

The Prognostic Value of FET PET in Glioblastoma

**Corresponding author:**

Sidsel Højklint Poulsen, Department of Radiation Biology, The Finsen Center, Section 6321, Rigshospitalet, Blegdamsvej 9, DK-2100 Copenhagen, Denmark; Tel: (+45) 35 45 63 27,

Email: sidsel.hoejklint.poulsen@regionh.dk

**Supplementary material**

**Tabel S1** – **Complete** **per** **patient data**

| **Clinical Characteristics** | | | | | | | | **FET PET Measures** | | | | | **Histological Biomarkers** | | **Endpoints** | |
| --- | --- | --- | --- | --- | --- | --- | --- | --- | --- | --- | --- | --- | --- | --- | --- | --- |
| **ID** | **Gender** | **Age** | **PS** | **GD+MRIvol** | **Use of corticosteroids** | **Multifocal Disease** | **Frontal location** | **TBRmean** | **TBRmax** | **BTV (1.6)** | **BTV (1.8)** | **BTV (2.0)** | **MGMT**  **Protein** | **IDH1 mutation** | **PFS** | **OS** |
| 1 | F | 65 | 2 | 10.00 | No | Yes | No | 1.91 | 2.76 | 8.24 | 4.61 | 2.34 | Neg | N/A | 9 | 9 |
| 2 | M | 26 | 0 | 7.70 | No | No | Yes | 1.92 | 3.35 | 6.4 | 2.67 | 1.69 | Pos | Neg | 38 | 38 |
| 3 | M | 62 | 1 | 6.80 | No | No | No | 1.82 | 2.53 | 12.4 | 5.4 | 1.8 | Pos | Neg | 4 | 7 |
| 4 | M | 38 | 0 | 5.20 | No | No | Yes | 1.88 | 2.66 | 37.55 | 20.12 | 9.9 | Neg | Pos | 17 | 17 |
| 5 | F | 54 | 0 | 9.60 | No | Yes | Yes | 1.83 | 2.63 | 15.25 | 7.28 | 2.28 | Neg | Neg | 11 | 20 |
| 6 | M | 67 | 1 | 0.00 | No | No | No | 1.69 | 1.9 | 3.25 | 0.3 | 0 | Neg | N/A | 5 | 14 |
| 7 | M | 65 | 1 | 0.00 | No | No | No | 1.69 | 1.86 | 3.58 | 0.16 | 0 | Pos | Neg | 7 | 10 |
| 8 | M | 50 | 0 | 0.00 | No | No | No | 1.83 | 2.55 | 1.9 | 0.84 | 0.33 | Pos | Neg | 10 | 25 |
| 9 | F | 71 | 1 | 0.00 | Yes | No | Yes | 1.73 | 2.09 | 2.3 | 0.51 | 0.08 | Pos | Neg | 10 | 16 |
| 10 | M | 43 | 0 | 0.00 | Yes | No | No | 1.79 | 2.32 | 7.74 | 2.9 | 0.86 | Pos | Neg | 6 | 16 |
| 11 | M | 55 | 0 | 0.00 | No | No | No | 1.73 | 2.15 | 8.53 | 2.18 | 0.23 | Pos | Pos | 11 | 19 |
| 12 | M | 51 | 0 | 0.00 | Yes | No | No | 1.78 | 2.36 | 9.23 | 3.3 | 0.76 | Pos | Neg | 4 | 17 |
| 13 | F | 33 | 0 | 0.00 | No | No | Yes | 1.75 | 2.21 | 4.89 | 1.3 | 0.48 | Neg | Neg | 23 | 30 |
| 14 | F | 59 | 1 | 0.00 | Yes | No | No | 0 | 0 | 0 | 0 | 0 | Neg | Neg | 10 | 35 |
| 15 | F | 50 | 0 | 0.00 | Yes | No | Yes | 1.65 | 1.79 | 0.49 | 0 | 0 | Neg | Pos | 14 | 27 |
| 16 | M | 47 | 0 | 0.00 | No | No | No | 1.68 | 1.87 | 1.4 | 0.08 | 0 | Neg | Neg | 7 | 20 |
| 17 | M | 66 | 0 | 0.00 | No | No | No | 1.7 | 2 | 1.68 | 0.13 | 0 | Neg | Neg | 14 | 16 |
| 18 | M | 47 | 1 | 0.00 | No | No | No | 1.76 | 2.28 | 1.69 | 0.59 | 0.13 | Neg | Neg | 9 | 19 |
| 19 | M | 70 | 0 | 0.00 | Yes | No | Yes | 1.69 | 1.88 | 2 | 0.12 | 0 | Neg | Neg | 19 | 19 |
| 20 | M | 43 | 0 | 0.00 | No | No | Yes | 1.69 | 2.05 | 2.28 | 0.3 | 0.02 | Neg | N/A | 34 | 34 |
| 21 | M | 61 | 0 | 0.00 | Yes | No | Yes | 1.72 | 2.07 | 3.8 | 0.62 | 0.05 | Neg | Neg | 17 | 22 |
| 22 | M | 34 | 0 | 0.00 | No | No | No | 1.72 | 2.03 | 3.96 | 0.69 | 0.02 | Neg | N/A | 22 | 37 |
| 23 | M | 67 | 0 | 0.00 | No | No | No | 1.78 | 2.17 | 4.23 | 1.59 | 0.32 | Neg | Neg | 4 | 10 |
| 24 | F | 62 | 0 | 0.00 | No | No | Yes | 1.75 | 2.16 | 5.86 | 1.82 | 0.15 | Neg | Neg | 11 | 17 |
| 25 | F | 46 | 0 | 0.00 | No | No | No | 1.76 | 2.13 | 5.88 | 1.89 | 0.26 | Neg | Neg | 4 | 12 |
| 26 | M | 64 | 0 | 0.00 | No | No | No | 1.94 | 3.16 | 9.12 | 5 | 2.97 | Neg | Neg | 9 | 13 |
| 27 | M | 50 | 0 | 0.00 | No | No | Yes | 1.75 | 2.31 | 10.06 | 2.68 | 0.69 | Neg | N/A | 6 | 14 |
| 28 | F | 71 | 1 | 0.00 | No | No | No | 1.74 | 2.06 | 16.26 | 4.6 | 0.19 | Neg | Neg | 7 | 24 |
| 29 | M | 65 | 0 | 0.00 | No | No | Yes | 1.89 | 2.65 | 17.18 | 10.08 | 4.75 | Neg | Neg | 7 | 16 |
| **30** | **M** | **59** | **1** | 0.00 | **Yes** | **No** | **Yes** | **1.93** | **2.79** | **25.84** | **15.71** | **8.81** | **Neg** | **Pos** | **4** | **13** |
| 31 | M | 58 | 0 | 0.00 | No | No | Yes | 1.97 | 3.8 | 29.65 | 18.35 | 11.11 | Neg | Neg | 18 | 18 |
| 32 | F | 77 | 1 | 9.80 | Yes | No | No | 1.97 | 3.02 | 21.67 | 13.72 | 8.34 | Neg | Neg | 4 | 10 |
| 33 | F | 65 | 1 | 14.40 | Yes | No | No | 1.92 | 3.25 | 33.07 | 17.93 | 10.85 | Neg | N/A | 3 | 12 |
| 34 | M | 50 | 0 | 17.20 | Yes | No | No | 1.84 | 2.28 | 13.6 | 0 | 0 | Neg | Neg | 4 | 7 |
| 35 | F | 71 | 0 | 6.40 | No | No | No | 1.85 | 2.82 | 24.6 | 10.38 | 5.15 | Neg | N/A | 7 | 15 |
| 36 | F | 54 | 1 | 0.50 | No | No | No | 1.85 | 3.01 | 31.71 | 14.26 | 5.94 | Neg | Neg | 7 | 12 |
| 37 | F | 53 | 2 | 17.60 | Yes | No | Yes | 2.46 | 4.35 | 35.27 | 28.43 | 23.58 | Neg | Neg | 2 | 8 |
| 38 | M | 42 | 1 | 29.30 | Yes | No | No | 2.41 | 5.91 | 96.86 | 71 | 53.2 | Neg | Neg | 4 | 11 |
| 39 | M | 36 | 0 | 8.40 | Yes | No | No | 2.05 | 3.79 | 38.94 | 26.99 | 18.44 | Neg | N/A | 4 | 13 |
| 40 | F | 69 | 1 | 18.00 | Yes | No | No | 1.81 | 2.82 | 10.11 | 3.32 | 1.67 | Neg | Neg | 2 | 9 |
| 41 | F | 53 | 1 | 10.80 | Yes | No | Yes | 2.32 | 4.33 | 46.27 | 36.39 | 28.82 | Neg | N/A | 4 | 18 |
| 42 | M | 63 | 1 | 22.90 | No | No | No | 2.24 | 3.88 | 38.72 | 30.26 | 23.85 | Neg | Neg | 12 | 23 |
| 43 | M | 58 | 1 | 112.30 | No | No | No | 1.82 | 2.55 | 10.5 | 4.23 | 1.74 | Pos | N/A | 10 | 13 |
| 44 | M | 56 | 1 | 20.60 | Yes | No | No | 2.14 | 3.72 | 34.88 | 24.7 | 17.48 | Pos | Neg | 5 | 37 |
| 45 | M | 59 | 1 | 4.60 | Yes | Yes | No | 2.14 | 3.34 | 54.12 | 41.51 | 30.08 | Pos | Neg | 4 | 4 |
| 46 | M | 56 | 0 | 6.50 | No | No | No | 1.75 | 2.25 | 4.6 | 1.27 | 0.24 | Pos | Neg | 7 | 12 |
| 47 | F | 67 | 0 | 8.80 | No | No | No | 2.13 | 4.67 | 16.11 | 8.93 | 6.53 | Pos | Neg | 2 | 7 |
| 48 | M | 53 | 0 | 11.80 | Yes | Yes | No | 1.81 | 2.36 | 24.32 | 10.56 | 3.75 | Pos | Neg | 4 | 15 |
| 49 | M | 69 | 0 | 4.40 | Yes | Yes | No | 2.07 | 3.96 | 27.83 | 18.11 | 12.53 | Pos | Neg | 19 | 27 |
| 50 | M | 52 | 0 | 12.90 | No | No | No | 2 | 3.75 | 28.21 | 17.48 | 10.13 | Pos | Neg | 3 | 20 |
| 51 | M | 62 | 0 | 27.10 | No | No | No | 2.04 | 3.56 | 45.48 | 27.75 | 18.87 | Pos | Neg | 4 | 6 |
| 52 | M | 59 | 0 | 7.60 | No | No | No | 1.68 | 1.88 | 1.13 | 0.08 | 0 | Pos | Neg | 6 | 12 |
| 53 | M | 68 | 1 | 4.50 | Yes | No | Yes | 1.72 | 2.08 | 10.71 | 2.29 | 0.05 | Pos | Neg | 6 | 9 |
| 54 | F | 70 | 0 | 0.60 | Yes | No | No | 1.92 | 2.6 | 14.76 | 9.16 | 4.94 | Pos | Neg | 7 | 13 |
| 55 | M | 65 | 1 | 1.90 | No | No | No | 1.79 | 2.43 | 18.66 | 6.98 | 2 | Pos | Neg | 23 | 32 |
| 56 | M | 65 | 1 | 11.70 | No | No | Yes | 1.85 | 2.85 | 18.69 | 8.37 | 3.76 | Pos | Neg | 4 | 18 |
| 57 | F | 72 | 0 | 6.40 | No | No | No | 2 | 4.16 | 38.92 | 25.37 | 15.18 | Pos | Neg | 4 | 15 |
| 58 | M | 61 | 1 | 14.10 | Yes | No | No | 1.9 | 3.19 | 52.4 | 29.05 | 14.56 | Pos | Neg | 9 | 12 |
| 59 | F | 67 | 0 | 1.60 | No | No | No | 2.12 | 4.07 | 68.84 | 47.07 | 33.15 | Pos | Neg | 4 | 20 |
| 60 | M | 65 | 1 | 5.80 | Yes | No | Yes | 1.78 | 3.36 | 7.46 | 1.85 | 0.69 | Pos | Pos | 4 | 11 |
| 61 | F | 61 | 0 | 26.60 | No | Yes | No | 2.21 | 3.98 | 16.18 | 11.32 | 8.75 | Pos | Neg | 4 | 8 |
| 62 | F | 72 | 0 | 18.80 | Yes | No | Yes | 1.94 | 3.35 | 21.32 | 11.57 | 5.68 | Pos | Neg | 14 | 14 |
| 63 | M | 66 | 0 | 7.50 | Yes | Yes | No | 2.5 | 6.12 | 22.99 | 16.34 | 12.91 | Pos | Neg | 4 | 10 |
| 64 | M | 78 | 1 | 7.70 | Yes | No | Yes | 2.02 | 4.33 | 33.39 | 21.3 | 13.17 | Pos | Neg | 12 | 20 |
| 65 | M | 64 | 1 | 48.10 | Yes | No | No | 1.97 | 3.79 | 71.53 | 39.78 | 24.11 | Pos | Neg | 9 | 10 |
| 66 | M | 55 | 1 | 40.90 | Yes | Yes | No | 2.16 | 3.36 | 82.03 | 62.13 | 47.29 | Pos | Neg | 2 | 4 |
| 67 | M | 56 | 1 | 55.40 | Yes | Yes | Yes | 2.31 | 5.24 | 84.48 | 58.06 | 42.54 | Pos | N/A | 3 | 4 |
| 68 | M | 50 | 0 | 73.60 | Yes | No | No | 2.22 | 4.32 | 137.71 | 103.83 | 77.13 | Pos | Neg | 9 | 12 |
| 69 | F | 47 | 1 | 1.40 | Yes | No | No | 2.1 | 3.14 | 6.6 | 4.64 | 3.39 | Pos | Neg | 13 | 16 |
| 70 | M | 78 | 0 | 7.00 | No | No | Yes | 1.82 | 2.74 | 29.89 | 13.77 | 4.35 | Pos | Neg | 6 | 19 |
| 71 | F | 58 | 0 | 14.30 | No | No | No | 1.87 | 2.87 | 31.28 | 16.39 | 7.42 | Pos | Neg | 4 | 11 |
| 72 | M | 54 | 2 | 6.60 | No | No | No | 1.93 | 3.2 | 39.55 | 23.85 | 13.19 | Pos | N/A | 4 | 11 |
| 73 | M | 52 | 1 | 7.10 | Yes | No | Yes | 2.05 | 4.15 | 45.89 | 26.55 | 17.31 | Pos | N/A | 8 | 15 |
| 74 | M | 61 | 0 | 13.50 | No | No | No | 1.99 | 5.29 | 48.24 | 25.07 | 15.42 | Pos | Pos | 4 | 28 |
| 75 | M | 71 | 1 | 28.90 | No | No | No | 2.05 | 5.14 | 64.15 | 38.9 | 24.47 | Pos | N/A | 11 | 12 |
| 76 | F | 52 | 0 | 36.60 | Yes | Yes | No | 2.14 | 3.81 | 105.76 | 82.27 | 61.35 | Pos | Neg | 6 | 16 |
| 77 | F | 69 | 1 | 6.80 | Yes | Yes | No | 2.25 | 5.32 | 13.83 | 9.02 | 6.8 | Neg | Neg | 20 | 33 |
| 78 | F | 66 | 1 | 16.50 | Yes | No | No | 2.19 | 4.45 | 78.52 | 55.74 | 41.17 | Neg | Neg | 4 | 6 |
| 79 | M | 66 | 0 | 8.10 | Yes | No | No | 1.74 | 2.01 | 1.31 | 0.36 | 0.02 | Neg | Neg | 21 | 21 |
| 80 | M | 69 | 0 | 3.70 | No | No | No | 2.68 | 4.99 | 8.18 | 6.36 | 5.57 | Neg | Neg | 22 | 25 |
| 81 | M | 59 | 1 | 3.00 | Yes | No | No | 1.78 | 2.56 | 8.24 | 3.07 | 0.59 | Neg | Neg | 8 | 14 |
| 82 | F | 56 | 0 | 5.50 | Yes | No | No | 2.06 | 4.27 | 9.74 | 5.81 | 3.84 | Neg | Neg | 9 | 14 |
| 83 | F | 61 | 0 | 7.40 | No | No | No | 1.95 | 2.77 | 11.42 | 7.29 | 4.11 | Neg | Neg | 18 | 29 |
| 84 | M | 60 | 1 | 10.10 | Yes | No | No | 1.8 | 2.51 | 16.82 | 6.48 | 2.78 | Neg | Neg | 4 | 13 |
| 85 | M | 40 | 1 | 21.30 | Yes | No | No | 1.77 | 2.17 | 18.76 | 6.96 | 0.66 | Neg | Neg | 5 | 21 |
| 86 | M | 67 | 1 | 9.80 | Yes | No | No | 1.84 | 2.63 | 21.87 | 10.5 | 4.29 | Neg | Neg | 1 | 1 |
| 87 | M | 68 | 0 | 22.10 | Yes | No | No | 1.78 | 2.72 | 28.2 | 8.83 | 3.41 | Neg | Pos | 17 | 17 |
| 88 | M | 65 | 1 | 9.20 | Yes | No | No | 1.83 | 2.7 | 28.35 | 13.42 | 4.68 | Neg | Neg | 5 | 12 |
| 89 | M | 48 | 1 | 8.70 | No | No | No | 1.93 | 3.13 | 31.22 | 17.67 | 9.6 | Neg | Neg | 7 | 12 |
| 90 | F | 64 | 1 | 21.00 | Yes | No | No | 2.23 | 3.78 | 71.52 | 54 | 41.85 | Neg | Neg | 2 | 3 |
| 91 | M | 69 | 1 | 12.80 | Yes | No | No | 2.61 | 5.63 | 79.28 | 64.73 | 54.83 | Neg | Neg | 3 | 12 |
| 92 | M | 56 | 0 | 39.60 | Yes | No | Yes | 2.09 | 3.27 | 82.8 | 60.22 | 42.51 | Neg | Neg | 3 | 9 |
| 93 | F | 42 | 0 | 3.50 | No | No | No | 1.64 | 1.77 | 0.66 | 0 | 0 | Neg | Neg | 12 | 12 |
| 94 | F | 49 | 0 | 0.80 | No | No | No | 1.65 | 1.77 | 0.66 | 0 | 0 | Neg | Neg | 7 | 25 |
| 95 | F | 69 | 0 | 2.50 | Yes | No | No | 1.67 | 1.88 | 2.01 | 0.07 | 0 | Neg | Neg | 5 | 18 |
| 96 | F | 60 | 0 | 5.00 | No | No | Yes | 1.75 | 2.12 | 4.97 | 1.56 | 0.19 | Neg | Neg | 10 | 21 |
| 97 | F | 70 | 0 | 18.40 | No | No | No | 1.77 | 2.29 | 7.06 | 2.37 | 0.52 | Neg | Neg | 35 | 35 |
| **98** | **M** | **70** | **0** | 3.20 | **No** | **No** | **No** | **1.77** | **2.25** | **7.76** | **2.83** | **0.52** | **Neg** | **Neg** | **31** | **31** |
| 99 | F | 67 | 1 | 1.60 | Yes | No | Yes | 1.71 | 2.06 | 11.31 | 2.18 | 0.09 | Neg | Neg | 7 | 10 |
| 100 | M | 77 | 0 | 0.00 | Yes | No | Yes | 1.87 | 3.32 | 12.85 | 6.56 | 2.74 | Neg | Neg | 14 | 17 |
| 101 | F | 34 | 0 | 1.60 | No | No | No | 1.79 | 2.2 | 13.64 | 5.61 | 1.24 | Neg | Neg | 22 | 23 |
| 102 | M | 54 | 0 | 7.80 | No | No | No | 2 | 4.21 | 14.37 | 7.7 | 4.33 | Neg | Neg | 9 | 16 |
| 103 | M | 68 | 1 | 5.50 | No | No | Yes | 1.81 | 2.44 | 20.22 | 8.94 | 3.08 | Neg | Neg | 9 | 12 |
| 104 | F | 59 | 1 | 25.70 | Yes | No | No | 1.8 | 2.37 | 23.47 | 9.87 | 2.51 | Neg | Neg | 4 | 14 |
| 105 | F | 58 | 0 | 13.50 | No | No | Yes | 2.28 | 4.48 | 24.63 | 16.84 | 11.93 | Neg | Neg | 4 | 17 |
| 106 | M | 59 | 0 | 23.10 | No | No | No | 1.76 | 2.45 | 28.72 | 8.98 | 1.77 | Neg | Neg | 4 | 22 |
| 107 | M | 58 | 0 | 13.40 | No | No | Yes | 1.87 | 2.77 | 37.6 | 19.42 | 8.88 | Neg | N/A | 14 | 30 |
| 108 | F | 55 | 1 | 19.60 | No | No | No | 1.94 | 2.7 | 45.8 | 30.09 | 17.15 | Neg | Neg | 7 | 12 |
| 109 | M | 58 | 0 | 7.90 | No | No | No | 2.46 | 5.65 | 50.94 | 39.32 | 31.12 | Neg | Neg | 7 | 12 |
| 110 | F | 64 | 1 | 15.10 | Yes | No | Yes | 2.01 | 3.56 | 55.16 | 33.52 | 21.1 | Neg | Neg | 6 | 9 |
| 111 | M | 72 | 1 | 8.90 | Yes | No | No | 1.76 | 2.59 | 5.83 | 1.51 | 0.59 | Neg | Neg | 14 | 18 |
| 112 | M | 54 | 0 | 4.40 | No | No | No | 1.92 | 3.36 | 13.22 | 6.68 | 3.66 | Neg | Neg | 8 | 12 |
| 113 | M | 45 | 0 | 10.60 | No | No | No | 2.53 | 4.6 | 27.62 | 22.77 | 18.88 | Neg | Neg | 4 | 20 |
| 114 | M | 71 | 0 | 18.20 | No | No | No | 2.06 | 3.54 | 51.99 | 34.11 | 22.57 | Neg | Neg | 4 | 20 |
| 115 | M | 46 | 0 | 57.30 | Yes | No | No | 2.01 | 3.26 | 92.42 | 64.05 | 40.27 | Neg | Neg | 4 | 24 |
| 116 | M | 51 | 0 | 42.50 | Yes | Yes | No | 2.46 | 5.7 | 98.19 | 73.43 | 57.55 | Neg | Neg | 4 | 13 |
| 117 | M | 63 | 2 | 122.40 | Yes | Yes | Yes | 2.09 | 3.73 | 102.43 | 71.02 | 50.23 | Neg | Neg | 1 | 1 |
| 118 | M | 49 | 0 | 78.90 | Yes | No | Yes | 2.71 | 5.89 | 154.86 | 127.15 | 105.52 | Neg | Pos | 7 | 14 |
| 119 | F | 66 | 1 | 8.60 | Yes | No | Yes | 1.75 | 2.19 | 4.25 | 1.18 | 0.27 | Neg | Neg | 3 | 3 |
| 120 | F | 66 | 1 | 4.30 | No | No | Yes | 1.71 | 2.01 | 5.46 | 1.06 | 0 | Neg | Neg | 16 | 16 |
| 121 | M | 53 | 0 | 0.00 | No | No | No | 1.83 | 2.74 | 6.97 | 2.95 | 1.36 | Neg | Neg | 5 | 18 |
| 122 | M | 49 | 0 | 25.90 | No | No | No | 1.76 | 2.24 | 13.63 | 4.42 | 0.57 | Neg | Neg | 31 | 31 |
| 123 | F | 70 | 1 | 15.60 | No | No | No | 2.04 | 3.79 | 19.91 | 10 | 6.91 | Neg | Neg | 4 | 14 |
| 124 | M | 50 | 0 | 5.80 | No | No | No | 2.09 | 3.4 | 25.33 | 18.16 | 12.32 | Neg | Neg | 7 | 13 |
| 125 | F | 53 | 1 | 19.10 | Yes | No | Yes | 2.09 | 3.63 | 26.73 | 17.47 | 12.17 | Neg | Neg | 15 | 21 |
| 126 | M | 67 | 2 | 12.90 | Yes | No | No | 1.78 | 2.81 | 36.17 | 11.7 | 4.27 | Neg | Neg | 11 | 21 |
| 127 | M | 79 | 0 | 20.70 | No | No | No | 1.87 | 2.86 | 36.7 | 18.1 | 8.46 | Neg | Neg | 6 | 19 |
| 128 | M | 57 | 0 | 27.90 | Yes | No | No | 2 | 3.22 | 52.55 | 33.25 | 21.66 | Neg | Neg | 8 | 8 |
| 129 | F | 72 | 0 | 7.10 | Yes | Yes | No | 1.98 | 2.66 | 15.3 | 10.96 | 7.03 | Neg | N/A | 14 | 14 |
| 130 | F | 69 | 0 | 15.50 | No | No | No | 2.36 | 5.17 | 61.58 | 46.6 | 37.07 | Neg | N/A | 4 | 4 |
| 131 | M | 70 | 2 | 39.20 | Yes | No | No | 1.83 | 2.71 | 22 | 0 | 0 | Pos | Neg | 1 | 2 |
| 132 | M | 59 | 0 | 12.70 | Yes | No | No | 2.37 | 4.61 | 31.9 | 24.39 | 19 | Pos | Neg | 8 | 14 |
| 133 | M | 76 | 0 | 17.00 | No | No | No | 2.53 | 4.75 | 35.87 | 29.34 | 24.44 | Pos | Neg | 4 | 5 |
| 134 | M | 71 | 1 | 22.20 | Yes | No | No | 2.31 | 4.07 | 46.61 | 36.88 | 29.5 | Pos | N/A | 4 | 7 |
| 135 | M | 63 | 1 | 25.60 | No | No | Yes | 3 | 6.94 | 72.47 | 61.76 | 54.27 | Pos | Neg | 5 | 5 |
| 136 | M | 68 | 1 | 41.10 | No | No | No | 2.41 | 4.53 | 134.86 | 110.13 | 90.76 | Pos | Neg | 3 | 3 |
| 137 | F | 77 | 1 | 19.50 | Yes | No | No | 1.69 | 2.1 | 6.5 | 0.84 | 0.04 | Neg | Neg | 4 | 9 |
| 138 | M | 70 | 0 | 2.80 | Yes | No | No | 2.37 | 4.49 | 14.47 | 11.3 | 8.97 | Neg | Neg | 4 | 9 |
| 139 | M | 60 | 2 | 15.70 | Yes | No | No | 1.93 | 2.74 | 17.89 | 11.23 | 6.52 | Neg | N/A | 10 | 17 |
| 140 | F | 58 | 0 | 13.70 | Yes | Yes | No | 2.07 | 3.3 | 37.76 | 24.22 | 16.23 | Neg | Neg | 4 | 18 |
| 141 | F | 47 | 2 | 23.50 | Yes | Yes | No | 2.09 | 3.73 | 55.99 | 37.52 | 25.36 | Neg | Neg | 4 | 4 |
| 142 | M | 51 | 0 | 36.00 | Yes | No | No | 2.02 | 3.18 | 68.41 | 44.41 | 28.21 | Neg | Neg | 25 | 29 |
| 143 | M | 52 | 2 | 18.00 | Yes | No | No | 2.68 | 5.47 | 69.92 | 56.32 | 46.82 | Neg | Pos | 12 | 13 |
| 144 | M | 54 | 0 | 23.60 | No | Yes | No | 2.34 | 4.04 | 70.06 | 58.3 | 47.99 | Neg | Neg | 2 | 20 |
| 145 | M | 60 | 1 | 30.30 | Yes | No | No | 1.92 | 2.98 | 80.17 | 47.46 | 24.54 | Neg | Neg | 5 | 8 |
| 146 | M | 76 | 1 | 25.10 | Yes | Yes | Yes | 2.31 | 4.32 | 83.43 | 62.22 | 49.24 | Neg | Neg | 4 | 10 |

Abbreviations: FET = *O*-(2-^18^F-fluoroethyl)-L-tyrosine positron emission tomography. TBR = tumor-to-background ratio, BTV = biological tumor volume, EGFR = epidermal growth factor receptor, MGMT = O(6)-methylguanine-DNA methyltransferase, IDH1 = isocitrate dehydrogenase 1, PFS = progression free survival, OS = overall survival, Gd+ MRIvol = contrast enhancing tumor volume on MRI, Pos = positive, Neg = negative.

| Tabel S2 - Univariate analysis | PFS  HR (95%CI) | OS  HR (95%CI) |
| --- | --- | --- |
| BTV (TBR>1.6) | 1.01 (1.01-1.02) P <0.001 | 1.01 (1.01-1.02) P <0.001 |
| BTV (TBR>1.8) | 1.02 (1.01-1.02) P <0.001 | 1.02 (1.01-1.02) P <0.001 |
| BTV (TBR>2.0) | 1.02 (1.01-1.03) P <0.001 | 1.02 (1.01-1.03) P <0.001 |
| TBR_mean_ | 3.10 (1.78-5.41) P <0.001 | 3.62 (1.84-7.10) P <0.001 |
| TBR_max_ | 1.33 (1.15-1.53) P <0.001 | 1.35 (1.14-1.60) P = 0.001 |
| Age (decades) | 1.12 (0.96-1.31) P = 0.136 | 1.36 (1.12-1.65) P = 0.002 |
| WHO PS |  |  |
| 1 vs. 0 | 1.71 (1.19-2.47) P = 0.004 | 2.39 (1.57-3.65) P <0.001 |
| 2 vs. 0 | 1.99 (0.98-4.02) P = 0.056 | 4.52 (2.19-9.32) P <0.001 |
| Use of corticosteroids ≥ 15mg/d |  |  |
| Yes vs. No | 1.46 (1.03-2.07) P = 0.035 | 1.99 (1.32-2.99) P = 0.001 |
|  |  |  |
| Contrast enhancing volume on MRI | 1.08 (0.99-1.18) P=0.082 | 1.17 (1.06-1.28) P<0.001 |
| MGMT protein |  |  |
| Positive vs. Negative | 1.70 (1.16-2.49) P = 0.006 | 2.34 (1.52-3-58) P < 0.001 |
| Multifocal |  |  |
| Yes vs. No | 1.68 (1.00-2.80) P = 0.048 | 1.72 (0.99-2.99) P = 0.053 |
| Gender |  |  |
| Female vs. Male | 1.04 (0.72-1.49) P = 0.844 | 1.02 (0.67-1.55) P = 0.923 |
| IDH1^#^ |  |  |
| Positive vs. Negative | 0.83 (0.38-1.78) P = 0.627 | 0.80 (0.32-1.99) P = 0.635 |
| P53 |  |  |
| Positive vs. Negative | 0.74 (0.50-1.09) P = 0.125 | 0.84 (0.54-1.32) P = 0.454 |
| EGFR |  |  |
| Positive vs. Negative | 1.31 (0.79-2.18) P = 0.297 | 0.97 (0.56-1.67) P = 0.907 |
| Deletion of 1p19q |  |  |
| Yes vs. No | 0.53 (0.24-1.15) P = 0.106 | 0.77 (0.31-1.90) P = 0.564 |
| Frontal location |  |  |
| Yes vs. No | 0.70 (0.47-1.05) P = 0.085 | 0.97 (0.62-1.52) P = 0.899 |
| Progression |  |  |
| Distant vs. Central | 0.92 (0.58-1.46) P = 0.724 | 1.37 (0.83-2.27) P = 0.220 |

*Evaluated on MRI, ^#^without secondary gliomas. Abbreviations: BTV = Biological Tumor Volume, TBR = tumor-to-background ratio, PS = performance status, MGMT = O(6)-methylguanine-DNA methyltransferase, IDH1 = isocitrate dehydrogenase 1, EGFR = epidermal growth factor receptor.

| **Prognostic group** |  |
| --- | --- |
| Age: 50  MGMT protein: negative  PS: 0 |  |
| Age: 50  MGMT protein: negative  PS: 1-2 |  |
| Age: 50  MGMT protein: positive  PS: 0 |  |
| Age: 50  MGMT protein: positive  PS: 1-2 |  |
| Age: 70  MGMT protein: negative  PS: 0 |  |
| Age: 70  MGMT protein: negative  PS: 1-2 |  |
| Age: 70  MGMT protein: positive  PS: 0 |  |
| Age: 70  MGMT protein: positive  PS: 1-2 |  |

**
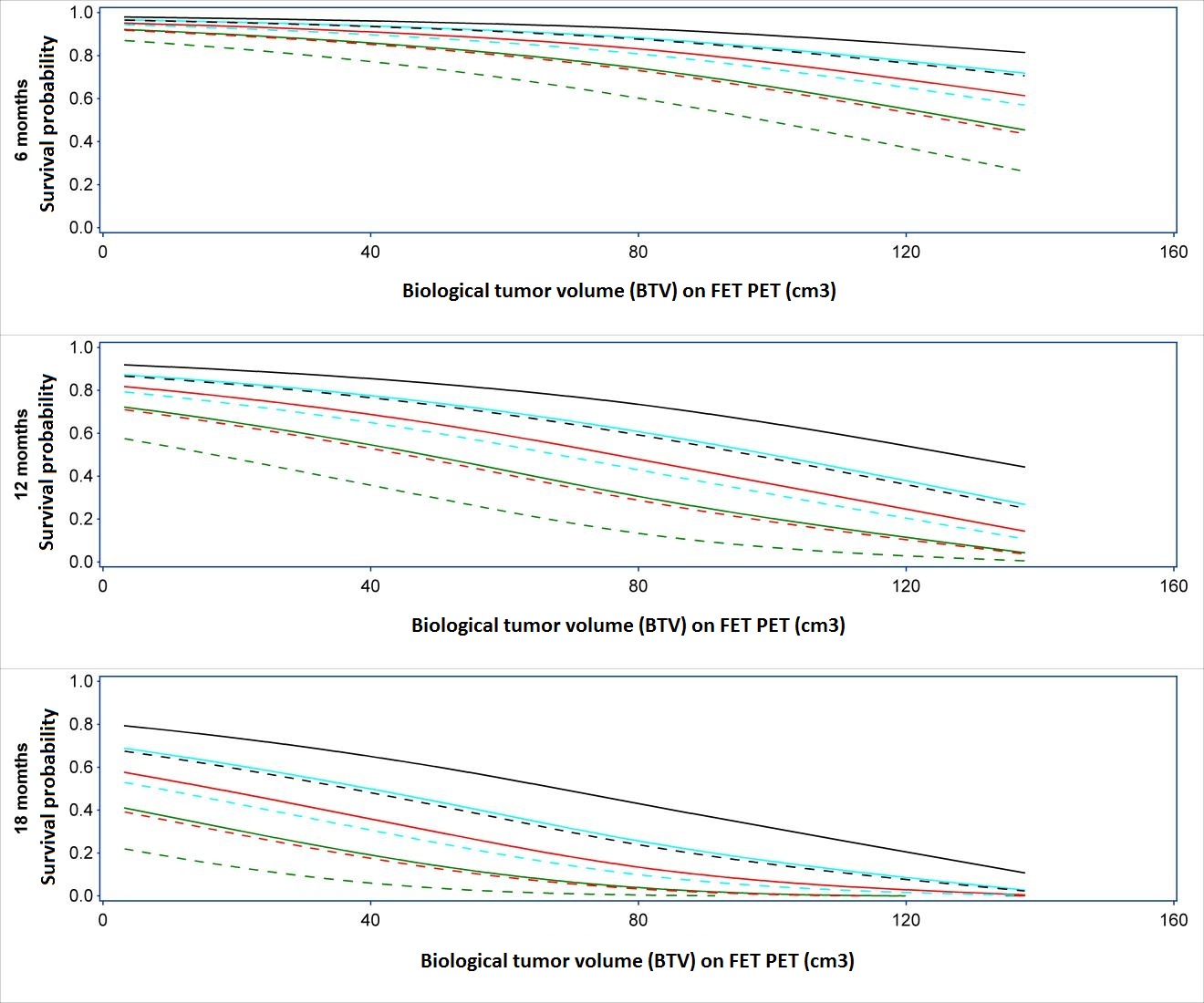
 Figure S2 – Prognostic model of survival probability as a function of biological tumor volume**

Survival probability at 6, 12 and 18 months as a function of biological tumor volume (BTV) derived from FET PET (> 1.6 B) for each prognostic group.


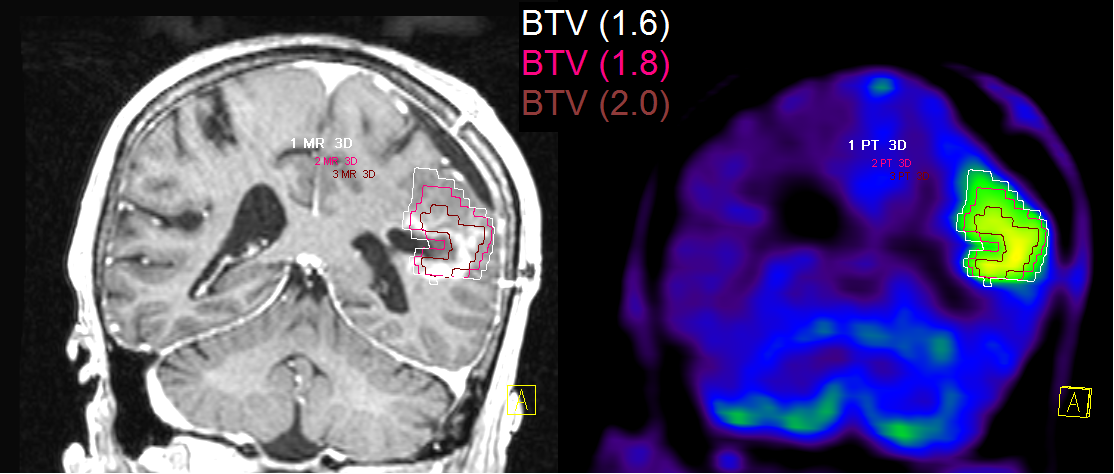
**Figure S1 - Definition of BTV on FET PET**

Left: Coronal section through GBM in left temporo-parietal region on contrast enhanced T1 weighted MRI of the brain 3 weeks postoperatively.

Right: Coregistered coronal FET PET scanning of same patient, showing increased activity uptake in tumor infiltrating surrounding tissue. Biological tumor volume (BTV) defined using 3 different tumor-to-background (TBR) are delineated (White:TBR > 1.6; Magenta: TBR > 1.8; Brown TBR > 2.0).

The left hemisphere is to the right of image.
